# Neurobehavioral correlates of inhibitory control in youth at-risk for early low-level alcohol use initiation: neuroimaging findings from the ABCD study

**DOI:** 10.3389/fpsyt.2026.1734436

**Published:** 2026-02-27

**Authors:** Faith Adams, Ahmet O. Ceceli, Siddhartha Peri, Iliyan Ivanov, Muhammad A. Parvaz

**Affiliations:** 1 Department of Neuroscience, Icahn School of Medicine at Mount Sinai, New York, NY, United States; 2 Department of Psychiatry, Icahn School of Medicine at Mount Sinai, New York, NY, United States; 3 Drexel University College of Medicine, Philadelphia, PA, United States; 4 Department of Artificial Intelligence and Human Health, Icahn School of Medicine at Mount Sinai, New York, NY, United States

**Keywords:** adolescent & youth, adolescent brain cognitive development study, impulsvity, inhibitory control, stop signal paradigm, substance use

## Abstract

**Introduction:**

Adolescent alcohol experimentation is a rising concern given its links to future problematic drug use. Difficulty with inhibitory control (i.e., the ability to suppress unwanted behaviors) is a well-known risk factor for early alcohol use onset. Nevertheless, little is known about the neurobiology of inhibitory control during early development (i.e., preadolescence), especially in relation to minimal early low-level alcohol use. The current study will reveal neural and behavioral differences in inhibitory control that differentiate youth will go on to engage in low-level alcohol experimentation compared with youth who remain alcohol naïve.

**Methods:**

The current study examined 80 pairs of demographically and developmentally matched youth from the Adolescent Brain Cognitive Development Study to predict early alcohol experimentation, consuming at least one full drink, but no regular use, prospectively (ages 10–14 years old). To identify the underlying neural mechanisms differentiating youth who endorsed alcohol experimentation (AE) and those who did not (AN), we utilized impulsive personality trait markers and neurobehavioral markers from the Stop Signal Task.

**Results:**

AE and AN youth showed no difference in task performance nor in impulsive personality traits but differed in patterns of neural engagement during the Stop Signal task. When compared to AN youth, AE youth displayed significantly higher activation in the right paracentral lobule and the left isthmus gyrus during the correct stop versus correct go contrast (indexing inhibitory control). Moreover, our findings indicated that, unlike in AN, a greater lack of planning in AE youth was associated with lower inhibitory control-related activation in the fusiform gyrus.

**Discussion:**

This study demonstrates a possible role of neural correlates of inhibitory control that are associated with substance use initiation. Despite behavioral similarities, the study detects differential neural markers of inhibitory control between AE and AN youth, an effect potentially driven by impulsive personality trait markers. As these markers could be both constitutionally and environmentally based, our results suggest that early substance use is accompanied by detectable differences in brain activation in key regions, which may be similar to those in later stages of use, highlighting the importance of delaying the age of alcohol onset.

## 1 Introduction

Adolescence is a complex transitional phase between childhood and adulthood, characterized by the rapid structural and functional reorganization of the brain (1). During this period, brain regions critical for decision-making and impulse control mature later than regions associated with emotions and reward processes, resulting in impaired inhibitory control, or the ability to control unwanted and inappropriate actions (2). This divergent period of maturation and compromised response inhibition is a risk factor for alcohol use (e.g., initiation, regular use, binge drinking, intoxication, and dependence) (3). While most neurodevelopmental studies examine brain changes during adolescence, these changes also occur during late childhood (ages 8–10 years old) (4), and risky behaviors begin to emerge (5), albeit in more subtle forms. Regardless of the expected nuances in the onset and development of maladaptive behaviors, the neurodevelopmental variability, i.e., ineffective engagement of inhibitory control circuits during this salient period, may confer risk for substance use involvement. As such, early examinations of brain mechanisms underlying inhibitory control may capture vulnerabilities that predispose youth to early alcohol use onset, with potential to add brain correlates to other tools aimed to inform timely and effective prevention and interventions.

Integrity of response inhibition is typically studied by quantifying stop signal reaction time via the Stop Signal Task (6) and commission errors via the Go/No Go Task, which are linked to alcohol dependence and binge drinking in youth (7). Studies of youth at a high risk for future onset have revealed altered neural trajectories in the fronto-basal ganglia network, parts of the default mode network, and in the prefrontal cortex, especially in the middle frontal gyrus, all before engaging in alcohol use (8). These findings suggest that there are mechanisms that may promote risk for initiation and subsequent escalation. Existing evidence regarding neural engagement associated with inhibitory control is inconsistent, as varying levels of substance use and the timing of risk assessment led to different neural activation patterns. For instance, youth who transition to heavy alcohol use demonstrate reduced activation in cognitive motor control regions, including frontal (e.g., pre-supplementary motor area), temporal, and parietal regions (9). Meanwhile, for other drinking patterns, neural engagement is reversed, with binge drinking youth showing greater activation in similar regions, primarily those in the prefrontal cortex (10). These different patterns of activations across similar degrees of substance use are apparent across other studies, albeit at different ages of risk assessment (9–11). Beyond neural mechanisms and behavioral aspects of impulsivity, individual differences are consistently linked to substance use risk, with traits such as impulsivity playing a key role in predicting early initiation and problematic use (12, 13). Uncovering the mechanisms underlying early adolescent substance use, impaired engagement of inhibitory control circuits, the inability to regulate impulsive responses, and trait-level dispositions during adolescence represents a crucial step towards understanding the neurodevelopmental risk factors involved.

Additionally, what constitutes alcohol naïve is not established across studies (14, 15), which is especially relevant as the neural substrates of alcohol initiation differ based on use patterns. For example, studies either collapse alcohol initiation (i.e., sipping or tasting) and experimentation (i.e., had a full drink) into a single binary outcome and compare it to alcohol naive youth, while some do not consider sipping as a meaningful alcohol use pattern and code them as alcohol naive (16, 17). However, with established evidence that differential neural engagement is associated with different patterns of substance use (18–20), it is critical that when assessing alcohol use, especially in younger adolescents, their drinking patterns are not conflated. In fact, there is little evidence of the neural correlates of inhibitory control that relate to minimal alcohol use (i.e., low-level experimentation before youth transition to regular use), since most studies have focused on youth with problematic substance use (9, 10, 21, 22).

Leveraging the Adolescent Brain Cognitive Development (ABCD) study (23), which collects neuroimaging data at baseline when participants are age 9–10 years old (an important salient period (24)) and then every two years thereafter, allows for understanding the adolescent alcohol use prospectively throughout adolescence, the most vulnerable window for sensitivity to alcohol use. As such, for the current analyses, we investigated the neural and behavioral markers of inhibitory control as well as impulsive-personality trait markers that differentiate alcohol-naïve youth who will prospectively go on to engage in low-level alcohol experimentation (i.e., endorsing having at least one full alcoholic drink, but no regular use, AE) from a demographically and developmentally matched sample of youth who did not go on to engage in alcohol experimentation (alcohol naive; AN). Here, we focused on three main hypotheses: compared to AN youth, 1) AE youth would exhibit less optimal inhibitory control and greater trait impulsivity, 2) AE youth would show increased neural activity in key cognitive control regions, including the presupplementary motor area, prefrontal cortex, as well as inhibitory control regions associated with the default mode network, and 3) AE youth are expected to exhibit greater activation during inhibitory control, which is associated with poorer performance and higher impulsivity.

## 2 Materials and methods

### 2.1 Participants

We leveraged data from the ABCD Study (Release 5.1). Participants (11,876 adolescents aged 9–10 years old) were recruited across 21 assessment sites in the United States, and to date, have been tracked longitudinally up to ages 14–15 years old. Participants completed a comprehensive battery of questionnaires assessing demographics, cognition, and substance use patterns, and underwent brain magnetic resonance imaging (MRI) and cognitive testing. Informed consent was collected at each study visit (25).

Participants’ age, sex, and race/ethnicity were collected at the baseline interview, and pubertal status (i.e., perceived physical maturation) was measured using the Pubertal Development Scale (PDS) (26). A combined score from the caregiver and youth reports was derived by averaging parent- and youth-reported pubertal status, with higher scores indicating more advanced pubertal development. These factors were used to identify a suitable non-using group comparison for AE youth, using the “MatchIt” R package (27).

**Figure 1:**
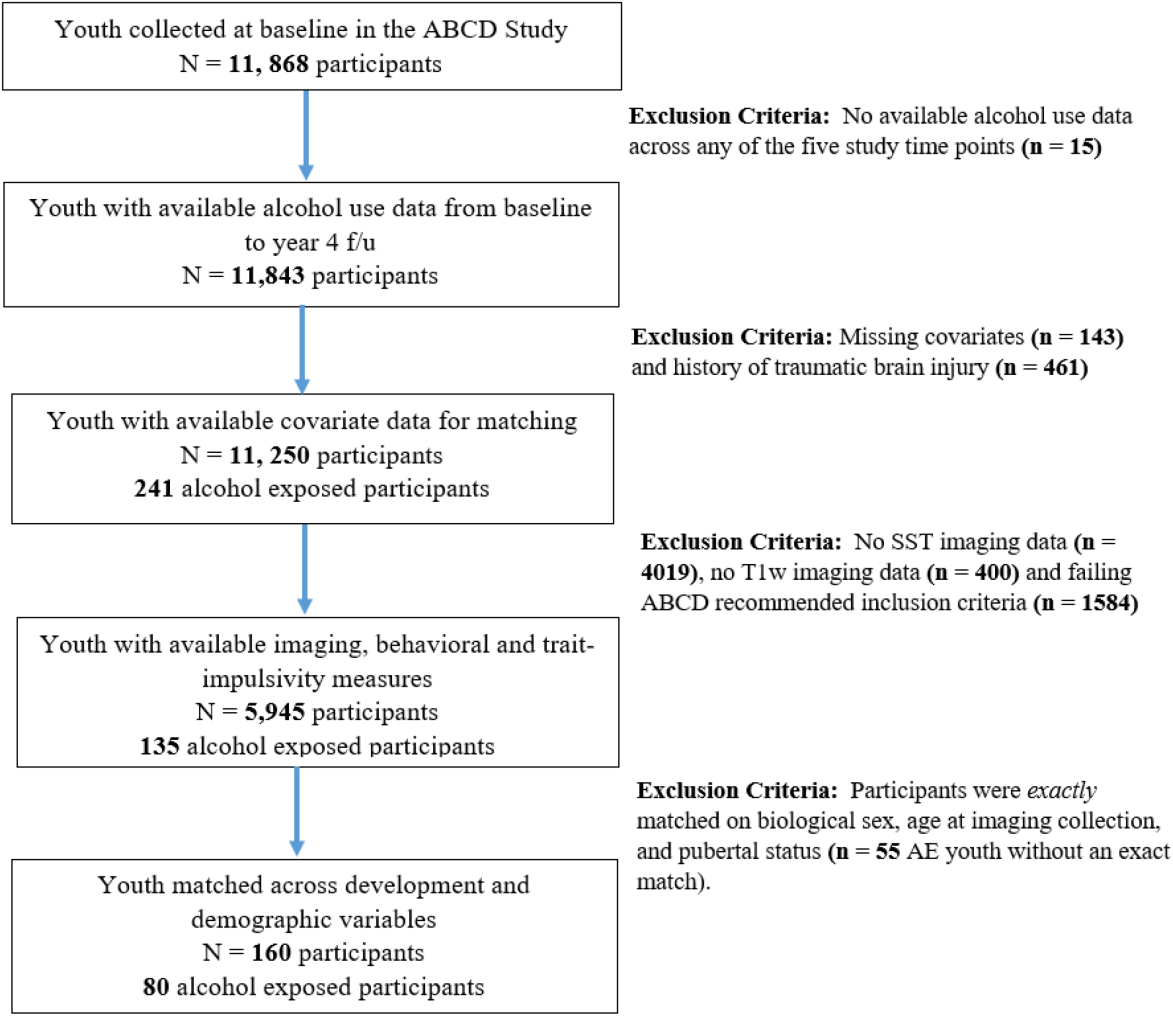
Flowchart of study sample.

For inclusion, participants were required to meet the recommended criteria for imaging (i.e., fMRI task-based SST and T1-weighted images at baseline) and behavioral data (described below in the experimental design and statistical analyses section); have no prior incidence of mild traumatic brain injury, as it is linked to deficits in inhibitory control (28); have complete information for the matching criteria variables (i.e., age, sex, race/ethnicity and pubertal status) and have an exact case-control match based on these variables. With this, we retained 80 pairs of age-, sex-, race/ethnicity- and pubertal status- matched youth with high-quality imaging and behavioral data for analyses (**Table 1**, **Figure 1**). Note that only baseline SST-task-fMRI data were used whereas we prospectively grouped participants based on alcohol use (full drink) between ages 9 and 15 years.

**Table 1:** Sample characteristics.

	Alcohol-Naïve Youth (n = 80)	Alcohol-Exposed Youth (n = 80)
Biological Sex		
Male	35(44%)	35(44%)
Female	45(56%)	45(56%)
Puberty Score	2.13(0.683)	2.14(0.675)
Age at Baseline (*in months*)	123 (7.28)	124(6.30)
Race/Ethnicity		
Non-Hispanic White	57(71%)	57(71%)
Non-Hispanic Black	2(3%)	2(3%)
Hispanic	13(16%)	13(16%)
Other	8(10%)	8(10%)
Age of Alcohol Use Endorsement		13.39 (1.52)
Max Drinking Days		3.21 4.09)

Dashes in table represent “not applicable” as the alcohol use-related variables only pertain to the AE youth.

#### 2.1.1 Minimal alcohol experimentation

Youth lifetime alcohol use was assessed longitudinally from baseline (ages 9-10) through Year 4 (ages 14-15) at annual in-person visits and biennial mid-year phone interviews. We were primarily interested in low-level alcohol experimentation before transitioning to regular use. Participants who would go on to consume a *full* drink of alcohol were categorized as alcohol exposed (AE), and those who did not consume alcohol were categorized as alcohol naïve (AN). Note that 75 of the 80 AE were alcohol-naïve at baseline, and the 5 who reported use at baseline had reported an average past year drinking of 1 day. We restricted comparisons to non-religious alcohol use; as such, youth who used alcohol within a religious context were coded as missing (15). Within the sample, the average number of drinking days within the past year specifically associated with the time of substance use onset was approximately 3.21 days. AE and AN youth were matched based on age-, sex-, race/ethnicity-, and pubertal status- matched youth. In our primary analyses, we examined the full sample of youth (i.e., 80 pairs). However, as a secondary analysis, we addressed the potential for reverse causation bias (29) and examined a sample excluding AE youth whose first use occurred at baseline when imaging data were collected (n = 5) and their matched AN pairs (n = 4) (see **Supplementary Material; Article I**).

### 2.2 Experimental design and statistical analysis

#### 2.2.1 The stop signal task

Participants underwent the SST paradigm which was used to assess neural activity during inhibitory control processes (6). During SST, participants are required to withhold or interrupt a response to a “Go” stimulus when presented with an unpredictable signal to stop. The task involves two runs containing 180 trials each, with each trial lasting 1000 milliseconds (ms). During each run, there are 30 stop trials in which the leftward and rightward facing arrows are replaced by the presentation of an upright arrow for 300ms and 150 trials. To ensure there are approximately 50% successful and 50% unsuccessful inhibition trials for Stop trials, a tracking algorithm varies the interval between the onset of the Stop Signal (Stop Signal Delay: SSD). The initial SSD is 50ms. Following an unsuccessful inhibition, the task is made easier by reducing the SSD by 50ms on the subsequent Stop trial. Following a successful inhibition, the task is made more difficult by increasing SSD by 50ms on the subsequent Stop trial.

The ABCD Study consortium conducted an initial assessment of the data quality, which found some issues with data, including overall task design and coding errors that impacted a small subset of subjects (30), which were corrected by applying trial-level and subject-level exclusion criteria (31) in the newest release (Release 5.1). Our analyses are conducted on data from Release 5.1. As per ABCD Study recommendations, we only included participants who met all criteria: SST*-*task-fMRI and T_1_ series passed raw quality control; task had no glitches; degrees of freedom exceed 200; E-prime timing matched or mismatch was acceptable; B0 field distortion corrected; FreeSurfer and fMRI post-processing QC passed, registration to T1w was acceptable and the dorsal/ventral field of view cutoffs were within limits (i.e., “imgincl_sst_include = 1”).

### 2.3 fMRI data acquisition and preprocessing

Images were acquired at 21 assessment sites using 3T MRI scanner platforms: Siemens Prisma, General Electric 750, and Phillips. The scanning parameters involved 90 x 90 x 60 slices, a field of view of 216 x 216, an echo time (TE)/repetition time (TR) of 800ms/30 ms, a flip angle of 52°, and a voxel resolution of 2.4 x 2.4 x 2.4 mm. Multiband echo planar imaging (EPI) with a slice acceleration factor was employed for the acquisition of scans (23). In ABCD, participants complete two other tasks (monetary incentive delay and emotional n-back task); therefore, the task order for each participant was randomized, except for participants from the same families. The total fMRI acquisition time was approximately 2 hours.

All fMRI data were minimally preprocessed by the ABCD Data Analysis, Informatics, and Resource Center (DAIRC) using published pipelines. In brief, structural scans were corrected for gradient non-linearity distortions, and initial rigid body alignment of T2-weighted images (T2w) to an atlas, followed by mutual information-based alignment to T1-weighted (T1w) images, and intensity non-uniformity correction through tissue segmentation and sparse spatial smoothing. The fMRI data were converted from raw to compressed files, distortion corrected using field maps, movement corrected, and aligned to standard space. Additionally, nuisance regressors, including motion estimates and physiological noise, were filtered and tabulated for researchers with the six motion regressors. Further details have been previously described elsewhere (32).

The structural and functional data were downloaded from ABCD and were further preprocessed using SPM12 (Statistical Parametric Mapping; http://fil.ion.ucl.ac.uk/spm/). As recommended by ABCD, we removed the initial scans to allow the fMRI signal to reach steady-state magnetization and ensure consistent voxel-wise normalization (32). First, we computed a mean functional image of all images in the time series, creating a stable, noise-reduced image. Next, we coregistered each participant’s corresponding T1w structural scan to their mean functional image and all volumes of the functional scan using mutual information. Next, we segmented the coregistered image into gray matter, white matter, and cerebrospinal fluid using tissue probability maps biased towards the standard Montreal Neurological Institute (MNI) space. For spatial normalization, we used the resulting forward deformation field to transform and warp functional data and volumes. We resampled the functional data to 3 x 3 x 3mm voxels, and as a final step, we smoothed the data using a 6 mm full-width-at-half-maximum isotropic Gaussian kernel to improve signal to noise ratio (33, 34).

### 2.4 Data analysis

We compared behavioral performance in the stop signal task and impulsive personality traits between groups using Welch’s two-sample t-tests, correcting for multiple comparisons using false discovery rate independently within each domain with a threshold of significance after correction set at p < 0.05.

#### 2.4.1 Behavioral impulsivity

Participants performance leading to exclusion from the current study were determined by the following criteria: fewer than 150 Go trials, less than 60% correct on Go trials, incorrect Go trials greater than 30%, late Go trials (across correct and incorrect trials) greater than 30%, no response on Go trials than 30%, fewer than 30 Stop trials and Stop trial accuracy lower than 20% or greater than 80%, as done in previous ABCD studies (35).

We leveraged numerous indices reflective of processes related to inhibitory control. These included stop signal reaction time (SSRT), commission error rates, and D’. SSRT, the time required to inhibit a motor response, was computed by subtracting the median SSD of all stop trials from the *n*th percentile Go reaction time, where *n* represents the percentage of successful inhibitions (6, 36), with higher SSRT representing slower stopping latency (or worse performance). Commission error rate, on the other hand, is the rate of erroneous performance by pressing the key when a stop signal is present and was calculated as the proportion of the incorrect stop trials relative to total stop trials. D’, a signal detection-like measure reflecting participants’ sensitivity to distinguishing targets and nontargets was calculated by Z-transforming hits (correct responses on Go trials) and false alarms (responses made on Stop trials) (37). As an example, poorer task performance would be represented by higher SSRT, elevated commission errors, and lower D’. Behavioral indices assessed included the stop accuracy, go accuracy, SSRT, SSD, go RT, and D’, commission errors.

#### 2.4.2 Trait impulsivity

Trait impulsivity was assessed using the Children-Short Form UPPS-P measure (35). This abbreviated 20-item questionnaire is used to measure five dimensions of impulsivity, including negative urgency, lack of perseverance, lack of premeditation, sensation seeking, and positive urgency.

### 2.5 BOLD fMRI data analyses

We distinguished four trial types: correct go (successfully going), correct stop (successfully withholding response after stop signal), incorrect stop (failing to inhibit responses after stop signal), and incorrect go (incorrect response to go trials). We modeled the BOLD signal by convolving the onset of each trial with the canonical (double gamma) hemodynamic response (38). Temporal derivatives, motion parameters (three translational and three rotational), and incorrect go trials were modeled as covariates of no interest. Since we discarded the first ten acquisitions during preprocessing, we adjusted the event-related data to remove the initial 8 seconds of data corresponding to those scans. The data were high-pass filtered (1/128 Hz cutoff) to remove low-frequency signal drifts. The final general linear model (GLM) was used to estimate the beta estimates representing each regressor’s fit to a voxel time series. Effects were estimated at each voxel for each run and resulting contrast maps for Correct Stop vs Correct Go contrast were then averaged across runs to yield subject-level contrast maps. This contrast (Correct Stop vs Correct Go) has been previously examined across the ABCD sample (n = 5,457), revealing consistent cluster activations in classical regions related to inhibitory control (35, 39).

Using the activation maps from our subject-level GLM, we conducted three main analyses to estimate group-level inhibitory control neural activations: 1) one sample t-test to identify BOLD signal across all participants, 2) two sample t-test to compare BOLD signaling differences between AE and AN youth, and 3) whole brain correlations between brain activation and behavior (i.e., SSRT, commission errors, D’, and UPPS-P measures), to examine differences in these relationships between AE and AN youth, all while using a predetermined starting voxel-wise threshold of *p*_uncorrected_ < 0.005 (T > 2.6) and cluster threshold of *p*_FWE_ < 0.05 for each test to balance both Type I and Type II errors (40). We conducted these analyses using separate higher-level GLM analyses to avoid multicollinearity (corrected for familywise error, α = 0.05/5 = 0.01).

To illustrate the magnitude and direction of activation, parameter estimates of peak activation were extracted from spherical (4-mm diameter) regions of interest from each participant using MarsbaR ROI toolbox (http://marsbar.sourceforge.net/) (41), and subsequent *post hoc* Pearson’s correlations and visualizations were conducted using R. For all analyses, values that were 3 SDs above or below the mean were identified as outliers and removed.

### 2.6 Covariates

Although participants were matched on race/ethnicity, age, biological sex, and puberty score, for our whole brain analyses, we only adjusted for age, sex, and pubertal status to account for their potential known influence on alcohol use and inhibitory control (42–44). We did not covary for race/ethnicity, since it is not known to be associated with inhibitory control (45). However, we conducted supplemental analyses including race as an additional covariate (see **Supplementary Material; Article II**). Moreover, although data were collected from multiple sites (n =22), and there is potential for scanner effects (46), we did not include scanner site as a covariate as it requires dummy coding (i.e., adding 22 covariates), increasing the model’s complexity. As a sensitivity analysis, we conducted an ROI-level analysis, accounting for scanner site as a random effect using a mixed-effects logistic model (see **Supplementary Material; Article III**). Beyond these potential confounders, the social environment is known for its role in alcohol initiation (47). However, to maintain the integrity of our statistical analysis (specifically, avoid losing degrees of freedom), we first analyzed major social characteristics to assess whether there were significant differences between alcohol-experimentation groups. Only those that show significant differences would be included as confounding variables in our final models. (see **Supplementary Material; Article IV**).

## 3 Results

### 3.1 Participant characteristics

Our study sample consisted of 80 pairs of AN and AE youth (n = 160 youth) (**Table 1**). Since participants were matched on age at baseline data collection, sex assigned at birth, pubertal status, and race/ethnicity, the groups were comparable on these factors (**Table 1**). Within the AE youth, the average age of alcohol use endorsement was 13.39 years old, and youth had an average of 3.21 drinking days over the past year (**Table 2**).

**Table 2:** Group differences in behavioral and trait impulsivity, with multiple comparisons correction applied separately within behavioral and trait impulsivity domains).

	AN (n = 80)	AE (n = 80)	Welch’s t-test	Effect size (Cohen’s d)
Correct Go RT (ms)	536(76.5)	522 (78.3)	*t*_(157.91)_ = 1.11, p = 0.268	0.176
Incorrect Stop RT (ms)	160 (31.9)	167 (32.1)	*t*_(158)_ = -1.34, p = 0.183	0.211
Stop Trial Accuracy (%)	51.4(5.92)	50.9 (5.49)	*t*_(157.11)_ = 0.58, p = 0.565	0.091
Go Trial Accuracy (%)	88.5 (7.37)	87.8 (7.92)	*t*_(157.19)_ = 0.52, p = 0.602	0.082
Target Sensitivity (D’)	1.97 (0.46)	1.94(0.54)	*t*_(153.86)_ = 0.28, p = 0.78	0.044
SSRT (ms)	295 (68.7)	307 (60.2)	*t*_(155.3)_ = -1.18, p = 0.239	0.187
Commission Error (%)	44.7 (8.68)	46.5 (6.77)	*t*_(149.13)_ = -1.51, p = 0.134	0.238
Lack of Perseverance	7.09 (2.55)	7.2 (2.52)	*t*_(157.99)_ = -0.28, p = 0.779	-0.044
Lack of Planning	8.46 (2.92)	8.43 (2.51)	t_(154.65)_ = 0.09, p = 0.931	-0.014
Positive Urgency	7.80 (2.77)	7.85 (2.95)	t_(157.37)_ = -0.11, p = 0.912	-0.017
Negative Urgency	8.27 (2.81)	8.53 (2.85)	t_(157.97)_ = -0.59, p = 0.558	-0.093
Sensation Seeking	10.10 (2.45)	10.46 (2.73)	t_(156.19)_ = -0.88, p = 0.379	-0.140

### 3.2 Behavioral results

The Welch’s two-sample t-tests revealed that there were no significant differences in the inhibitory control behavioral performance of AE and AN youth. Moreover, we did not observe any differences in perseverance between groups (**Table 1**).

### 3.3 BOLD fMRI results

#### 3.3.1 Inhibitory control-related neural signaling

##### 3.3.1.1 Across all participants

**Figure 2:**
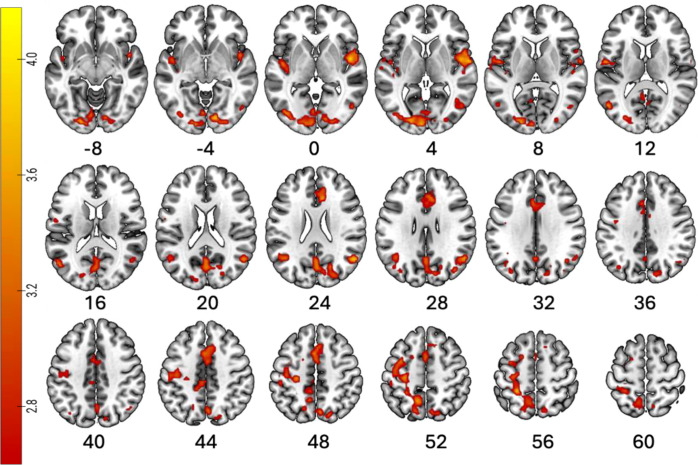
Whole brain correlates of inhibitory control across all participants. Activation maps displaying brain regions activated across all participants during inhibitory control, measured from the correct stop vs correct go contrast in the right superior temporal gyrus, right inferior parietal cortex, right caudal anterior cingulate cortex, lateral occipital cortex, and the precuneus cortex (**Table 3**). Significant results were detected using a cluster-defining threshold of T > 2.6, corrected to p_FWE_ < 0.05. The labels below each slice indicate the x-axis coordinates from the coronal sections in the MNI space from x = -8 to x = 60. The color bar represents the voxel T-value. Image orientation is neurological (i.e., Right = Right). Coordinates are in MNI-152 space. For labelling, data were resampled to an isotropic size of 2mm. R, right; L, left.

Whole brain analysis of inhibitory control across all participants (i.e., neural signaling during the Correct Stop > Correct Go contrast) yielded patterns of BOLD activation in the right superior temporal gyrus (MNI Space: 48, -1, 2; peak Z = 4.12; *p*_FWE_ = 0.028, 172 voxels), left inferior parietal cortex (MNI: 54, -61, 26; peak Z = 3.88, *p*_FWE_ = 0.035, 162 voxels), right caudal anterior cingulate cortex (MNI: 9, 26, 26; peak Z = 3.85, *p*_FWE_ = 0.002, 296 voxels), left lateral occipital cortex (MNI: -24, -88, -10; peak Z = 3.82, *p*_FWE_ = 0.000, 471 voxels), left superior parietal cortex (MNI: -27, -40, 56; peak Z = 3.73, *p*_FWE_ = 0.000, 480 voxels) and the right precuneus cortex (MNI: 6, -70, 47; peak Z = 3.48, *p*_FWE_ = 0.000, 372 voxels) (**Figure 2****) (****Table 3**).

**Table 3:** Inhibitory Control Brain Activity.

Region	L/R	p_FWE_	p_unc_	k	T	Z	x	y	z
All Participants									
Lateral Occipital Cortex	L	0.000	0.000	471	3.92	3.82	-24	-88	-10
Superior Parietal Cortex	L	0.000	0.000	480	3.82	3.73	-27	-40	56
Precuneus Cortex	R	0.000	0.000	372	3.56	3.48	6	-70	47
Caudal Anterior Cingulate Cortex	R	0.002	0.000	296	3.95	3.85	9	26	26
Superior Temporal Gyrus	R	0.028	0.002	172	4.24	4.12	48	-1	2
Inferior Parietal Cortex	R	0.035	0.002	162	3.98	3.88	54	-61	26
AE > AN									
Paracentral Lobule	R	0.000	0.000	452	3.59	3.52	6	-25	53
Isthmus Cingulate Cortex	L	0.032	0.002	160	3.62	3.54	-3	-49	32

Inhibitory control brain activity (Correct Stop > Correct Go) across all participants, within AE youth, and between-group differences.

##### 3.3.1.2 Group differences in inhibitory control-related neural signaling

**Figure 3:**
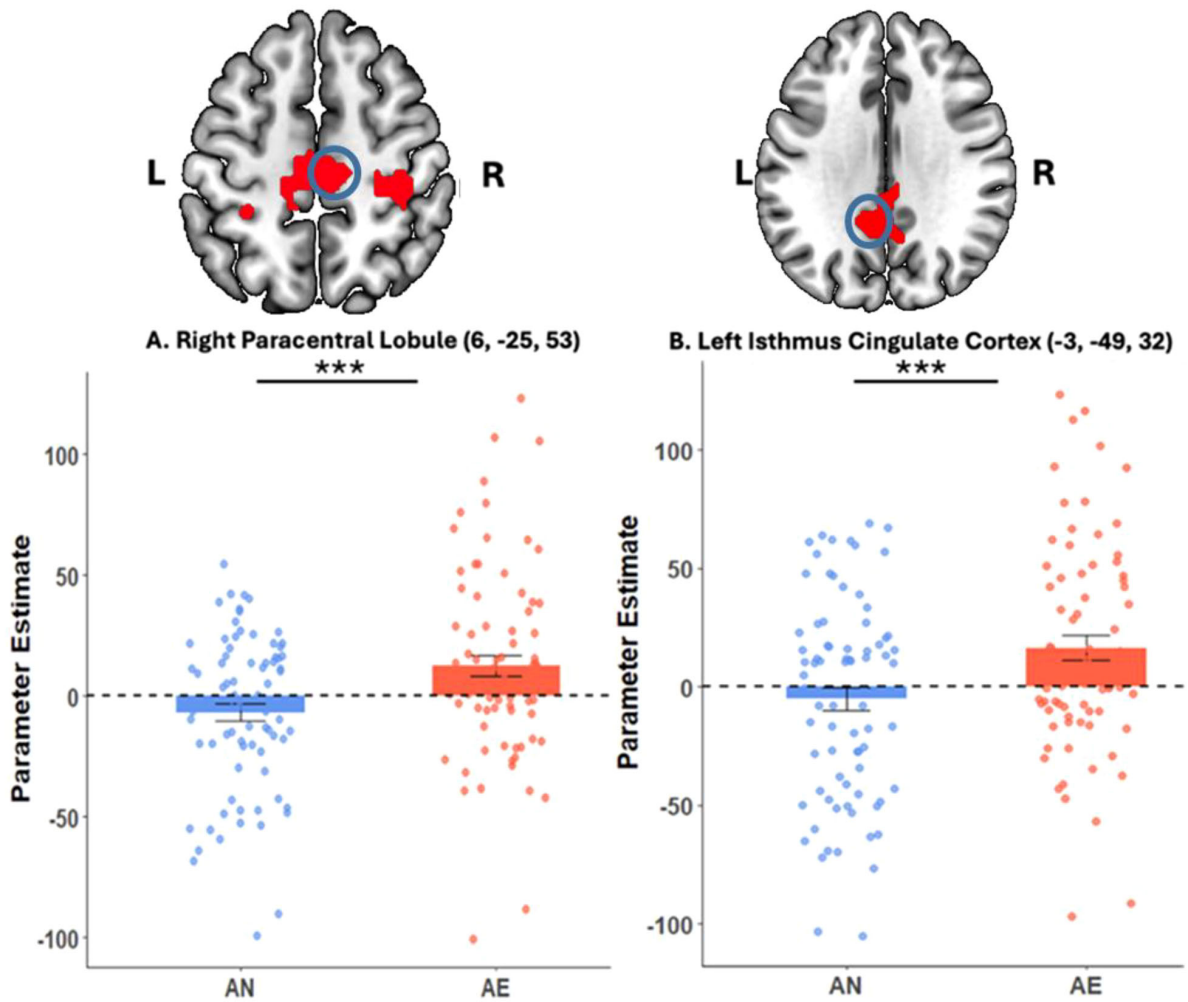
Group differences in neural activation. AN and AE youth differed in inhibitory control BOLD activation patterns. When compared to AN youth, AE youth displayed significantly higher activation in the right paracentral lobule **(A)** and the left isthmus cingulate cortex **(B)** during the correct stop vs correct go contrast. The color bar represents voxel T value. Circles denote peak voxels of the statistically significant clusters. Image orientation is neurological (i.e., Right = Right).

Whole-brain analysis of the overall inhibitory control between AN and AE youth revealed increased activation in the right paracentral lobule (MNI: 6, -25, 53; peak Z = 3.52, *p*_FWE_ = 0.000, 452 voxels) (**Figure 3A**) and the left isthmus cingulate cortex (MNI: -3, -49, 32; peak Z = 3.54, *p*_FWE_ = 0.032, 160 voxels) in AE youth compared to AN youth (**Figure 3B****) (****Table 3**). There were no significant clusters when comparing AN youth to AE youth.

#### 3.3.2 Whole brain correlations between brain and behavior

**Figure 4:**
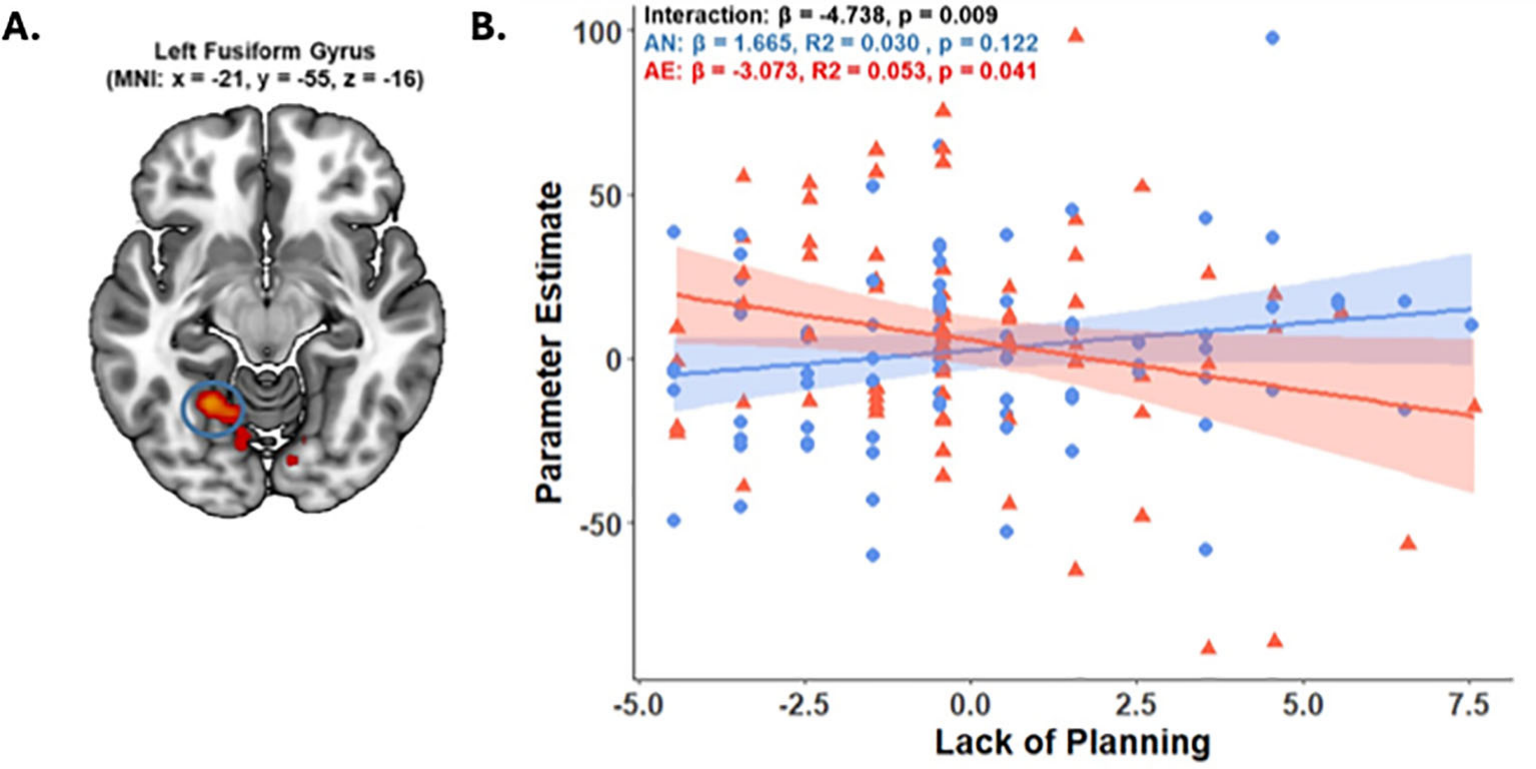
Brain-behavior correlation. Correlations with (lack) of planning in the left fusiform gyrus in AE and AN youth. **(A)** shows clusters of significant BOLD contrast (Correct Stop > Correct Go) in the left superior temporal gyrus, with blue crosshairs marking peak IC-related activity detected using a cluster-defining threshold of p < 0.005, p_FWE_ < 0.05. **(B)** displays the parameter estimates at the 4-mm sphere at voxel x = -21, y = -55, z = -16 for AE (n = 80, red triangles) and AN (n = 80, blue circles) youth; one outlier (± 3 SD) was excluded. The group interaction and the individual slopes of the AE and AN youth are presented in the figure.

Voxel-wise correlations between inhibitory control brain activity and behavioral (i.e., SSRT and d’) and personality (i.e., negative urgency, lack of perseverance, lack of planning, sensation seeking, positive urgency) measures yielded a significant difference in the relationship between the lack of planning, and AE and AN youth (p < 0.005, p_FWE_ < 0.05, k > 50 voxels). Specifically, we revealed a significant slope difference between groups: in the AE group, reduced neural activity in the left fusiform gyrus (BA 37; MNI space –21, -55, -16; peak Z = 4.00, p = 0.005, 244 voxels) was significantly correlated with a higher lack of planning (i.e., more impulsive) (**Figure 4**). In addition to the whole-brain correlations, we restricted the correlation analyses to the inhibitory control regions identified from the whole-brain analyses across all participants (3.3.1.1) (see **Supplementary Material; Article V**). However, this yielded no significant clusters.

## 4 Discussion

The current study aimed to investigate neural and behavioral differences in inhibitory control in a developmentally and demographically matched sample of alcohol-naïve youth, a subset of whom later engaged in low-level alcohol use experimentation (i.e., alcohol exposed, AE), and others who will continue to stay alcohol naïve (AN). Although our results showed no differences in behavioral performance of the SST, we observed significant between-group differences in the patterns of neural engagement in the right paracentral lobule and the left isthmus of the cingulate gyrus during inhibitory control. Moreover, we provided new evidence for a relationship between inhibitory control-related activation in the fusiform gyrus and lack of planning, differentiating AE and AN youth. Our study represents one of the earliest investigations of the relationship between neural correlates in alcohol-naïve youth and prospective onset of low-level alcohol use, utilizing imaging data collected in participants at ages 9–10 years old and assessing the prospective relationships in alcohol experimentation until the age of 14. Findings from this study advance our understanding of potential neurobiological mechanisms underlying inhibitory control predispositions associated with the earliest degrees of alcohol use before youth transition to problematic use.

Behaviorally, we anticipated differences in inhibitory control performance between AE and AN youth, as prior evidence suggests that impairments in inhibitory control precede alcohol use onset (48–50). Our results did not confirm this hypothesis; instead, we observed that the behavioral performance on the SST and trait-related impulsivity between groups were comparable. We speculate that perhaps the onset of low degree of alcohol use severity (51), even in those who initiated use later (i.e., youth endorsed having at least one full drink, with an average past year drinking of 3.21 days), contributed to the observation of comparable behavioral performance between the groups. Evidence from higher degrees of substance use, exceeding the average of the drinking days examined in this study, revealed similar inhibitory control behavioral patterns compared to non-users. Specifically, examinations of binge drinking (22.91 drinking days per month) (52), initiation of heavy drinking (2.8 drinking days per month) (9), alcohol misuse (3 lifetime binge drinking episodes by age 14) (53), and other patterns of drinking. While our study findings did not reveal specific behavioral differences, this corroboration with prior evidence from more severe patterns of substance use highlights that even when differences in behavioral mechanisms are not observed, there may still be underlying neural mechanisms that differentiate these youth.

From the current investigation, we did not observe significant effects in canonical cognitive control regions (e.g., dorsolateral prefrontal cortex or the anterior insula) (54, 55). Instead, across all participants, we identified effects in adjacent parietal and cingulate regions, as well as engagement in default mode and sensory-related areas. Moreover, AE youth recruited the paracentral lobule and the isthmus cingulate, regions linked to sensorimotor processing and default mode network activity, respectively. In fact, these findings are supported by prior evidence across various behavioral patterns, including substance use (10, 20, 39, 56, 57), emphasizing that with the ongoing maturation of the prefrontal regions, adolescents recruit broader or alternative areas to compensate.

Interestingly, in our study, AE youth showed greater engagement of the isthmus cingulate cortex related to inhibitory control, compared to AN youth. Though the isthmus cingulate cortex is not typically observed during inhibitory control, since it serves as a bridge between the posterior cingulate cortex and the parahippocampal gyrus, it supports visuospatial, contextual, and memory-related behaviors, and serves as a key node in the default mode network, which interacts with executive control systems (58, 59). In fact, adolescents’ transition to heavy drinking has previously been shown to be associated with smaller brain volumes and greater cortical thinning in the isthmus cingulate with increasing doses (60). Though converging, in other instances, studies of alcohol use (60), internet gaming disorder (61), and impulsive control disorder (62) found cortical thinning in risky groups compared to healthy controls. Moreover, an examination of sex-specific neural activation differences in the Go/No Go task in an adolescent population demonstrated that girls showed enhanced functional connectivity in the isthmus cingulate gyrus, among other regions of the default mode network, compared to boys (63). Although a sex-specific focus, this evidence highlights the relevance of the isthmus cingulate in an adolescent population.

Our research may reflect some compensatory processes involving the paracentral lobule’s role in inhibitory control before youth transition to more severe patterns of alcohol use. Corroborating evidence from two independent studies revealed that within high-risk youth (i.e., heavy prenatal alcohol exposure and family history of substance use), there is an intensified involvement of the paracentral lobule during response inhibition (20, 24). Indeed, low-level alcohol use represents a risk pattern like these, suggesting that at higher risk for future substance use, there is consistent recruitment of additional neural resources when learning, initiating, and inhibiting motor responses, compared to their low-risk counterparts. The recruitment of the paracentral lobule can be explained by age-related modulations (i.e., less recruitment for unrelated tasks with greater maturation) (64) and structural changes in response to impulsive behaviors (65). While functionally, there are maturation-related improvements in inhibitory control, diverging structural evidence of cortical thickness and poor performance within a high-risk sample raises a vital concern, as early initiation increases the likelihood of future problems.

One of the more notable findings from this study was the differential activation patterns of the left fusiform gyrus related to the lack of planning in AE and AN youth. We found that in youth who go on to engage in alcohol use, as their impulsive lack of planning increases, the activation of the fusiform gyrus decreases. Although these associations have not been previously elucidated, existing research suggests a potential explanation for this relationship. Evidence from a Tower of London Task, a task for assessing planning ability, found that the fusiform was primarily involved in the visuospatial processing, and subtle neural responses were also observed during planning (66). Furthermore, lesions in the fusiform gyrus were associated with greater global impulsivity (i.e., a general dimension of motor, non-planning, and attentional impulsivity) (18). Other investigations of low-level alcohol use in adolescents found that alcohol-exposed youth typically revealed more extensive activation patterns than nonusers in the fusiform gyrus (67), suggesting that the fusiform gyrus indeed plays a role in inhibitory control. Our study findings indicate that the neural activation in the fusiform gyrus and planning deficits in AE youth may make them more prone to atypical activation of this region, as if it were not functioning adequately.

The simultaneous engagement of the isthmus cingulate cortex and the paracentral lobule during SST offers some intriguing insights. While the activation of the isthmus cingulate may reflect atypical engagement due to its limited relevance to the inhibitory control processes, our findings indicated concurrent activation of the paracentral lobule in the AE group. This apparent activation suggests a potential compensatory reliance on sensorimotor processors, in the context of disrupted cognitive control network engagement, to maintain performance. Moreover, we observed engagement of another related posterior region, the fusiform gyrus, in response to higher impulsivity in AE youth. Together, these results highlight a reliance on less specialized neural networks during SST, a phenomenon that we can speculate is unique to adolescent populations due to a lack of maturation in the necessary brain regions (64, 68). Since this investigation is the first to differentiate between youth who later engage in low-level alcohol use and those who do not, using predisposing neural correlates collected at ages 9-10, future research is needed to further examine the observed associations and see if they hold in larger populations.

Important limitations are to be considered. In this investigation, we only examined 160 participants (n = 80 AE and n = 80 AN youth). However, this was a result of strict exclusion criteria (**Figure 1**), which ensured robustness and interpretability. Second, while our study is prospective, there were five participants out of 160 (less than 10%) who endorsed alcohol use at baseline imaging collection. To address any potential confounding bias, we conducted sensitivity analyses excluding baseline AE youth and their matched AN counterparts. As expected, there were no changes in behavioral performance between the groups. However, we observed differences in neural signaling compared to our primary analyses. Specifically, we observed significant cluster activations in the right postcentral gyrus and the left postcingulate cortex. Comparably, our primary analyses identified the isthmus cingulate, which links the posterior cingulate to the parahippocampal gyrus, and when using a different atlas (AAL), both regions overlapped. Most importantly, both regions were hyperactivated, as seen in our primary analyses. This suggests that these adolescents’ exposure to use at the same time as imaging collection did not modify activation patterns. Lastly, in our whole-brain analyses, we did not account for the variations in scanners across different sites, despite their importance (46, 69). We excluded this covariate because there were 24 unique scanners, and of the 24, there were five scanners without representation of either AE or AN youth, affecting model performance.

In summary, the findings of this study suggest that before the onset of even low-level alcohol experimentation and well before transitioning to regular use, there are existing neural differences that provide insight into inhibitory control brain activity in alcohol naïve and exposed youth, with engagement of sensorimotor and limbic regions. Further research in a larger sample is critical to interrogate and validate the compensatory mechanisms at play. Future studies will be instrumental in revealing whether the divergence in neural engagement during inhibitory control continues as youth progress to the next step in their alcohol use exploration.

## Data Availability

Publicly available datasets were analyzed in this study. This data can be found here: https://nda.nih.gov/abcd.
