# Supplementary material for: Neurobehavioral correlates of inhibitory control in youth at-risk for early low-level alcohol use initiation: neuroimaging findings from the ABCD study
Source: Front Psychiatry. 2026 Feb 27;17:1734436. doi: 10.3389/fpsyt.2026.1734436 (PMC12982181; doi:10.3389/fpsyt.2026.1734436)
Supplement: Supplementary file 1 [file DataSheet1.docx]

# Supplemental Material for “Neurobehavioral Correlates of Inhibitory Control in Youth At-Risk for Early Low-Level Alcohol Use Initiation: Neuroimaging Findings from the ABCD Study”

Faith Adams, PhD^1,2^, Ahmet O. Ceceli, PhD^2^, Siddhartha Peri, MS^3^, Iliyan Ivanov, MD^2^, Muhammad A. Parvaz, PhD

# **Handling Reverse Causation**

## **Methods**

Given the potential for reverse causation bias (46) (i.e., the effects of alcohol use on inhibitory control), we conducted all analyses described above, excluding AE youth whose first use occurred at baseline when imaging data were collected (n = 5) and their matched AN pairs (n = 4). Studies investigating the consequences of alcohol intake on inhibitory control primarily focus on heavy or binge drinking, and the patterns of alcohol use in our study may not be severe enough to biologically or behaviorally affect brain function and inhibitory control(47). It is important to note that during the GLM process, there was one participant without adequate data in their event file, and they were excluded from further analyses, and this turned out to be the AN match of one of the AE youth who initiated at baseline.

## **Results**

### **Participant Characteristics**

There were no changes in the matched demographic and developmental characteristics. Among our AE youth, the mean age of first-time alcohol use was endorsed at 13.69 years old (compared to 13.39 years old), with the earliest age of onset at 10.67 years old (compared to 9.25 years old in the primary analyses). Participants remained comparable between groups.

### **Behavioral Results**

There were no significant differences in behavioral performance between AE and AN youth.

### **BOLD fMRI Results**

#### **Across all participants**

Whole brain analysis of inhibitory control across all participants (i.e., neural signaling during the Correct Stop > Correct Go contrast) yielded patterns of BOLD activation in the left lateral occipital cortex (MNI: -24, -88, -10; peak Z = 3.96, *p*_FWE_ = 0.006, 282 voxels), left precentral gyrus (MNI: -30, -22, 53; peak Z = 3.78, *p*_FWE_ = 0.000, 254 voxels) and the right precuneus cortex (MNI: 6, -70, 47; peak Z = 3.37, *p*_FWE_ = 0.037, 191 voxels) **(Table S1)**.

| **Region** | **L/R** | **pFWE** | **p(unc)** | **k** | **T** | **Z** | **x** | **y** | **z** |  |
| --- | --- | --- | --- | --- | --- | --- | --- | --- | --- | --- |
| All Participants | | | | | | | | | | |
| Lateral Occipital Cortex | L | 0.006 | 0.000 | 282 | 4.08 | 3.96 | -24 | -88 | -10 |  |
| Precentral Gyrus | L | 0.011 | 0.001 | 254 | 3.78 | 3.68 | -30 | -22 | 53 |  |
| Precuneus Cortex | R | 0.037 | 0.003 | 191 | 3.45 | 3.37 | 6 | -70 | 47 |  |
| **AE > AN** | | | | | | | | | | |
| Postcentral Gyrus | R | 0 | 0 | 569 | 3.51 | 3.43 | 33 | -31 | 59 |  |
| Precuneus Cortex | R | 0.051 | 0.004 | 205 | 3.66 | 3.57 | 0 | -52 | 32 |  |

**Table S1: Inhibitory Control Brain Activity**.

Inhibitory control brain activity (Correct Stop > Correct Go) across all participants, within AE youth and between group differences removing participants who used at baseline and their matched counterparts.

#### **Group Differences**

Whole-brain analysis of the overall inhibitory control between AN and AE youth without baseline users revealed increased activation in the right precuneus cortex (MNI: 0, -52, 32; peak Z = 3.57, *p*_FWE_ = 0.05 205 voxels) **(Fig. S1, Panel A)** and the right postcentral gyrus (MNI: 33, -31, 59; peak Z =3.43, *p*_FWE_ < 0.001, 569 voxels) **(Fig. S1, Panel B)** in AE youth compared to AN youth **(Table S1)**.


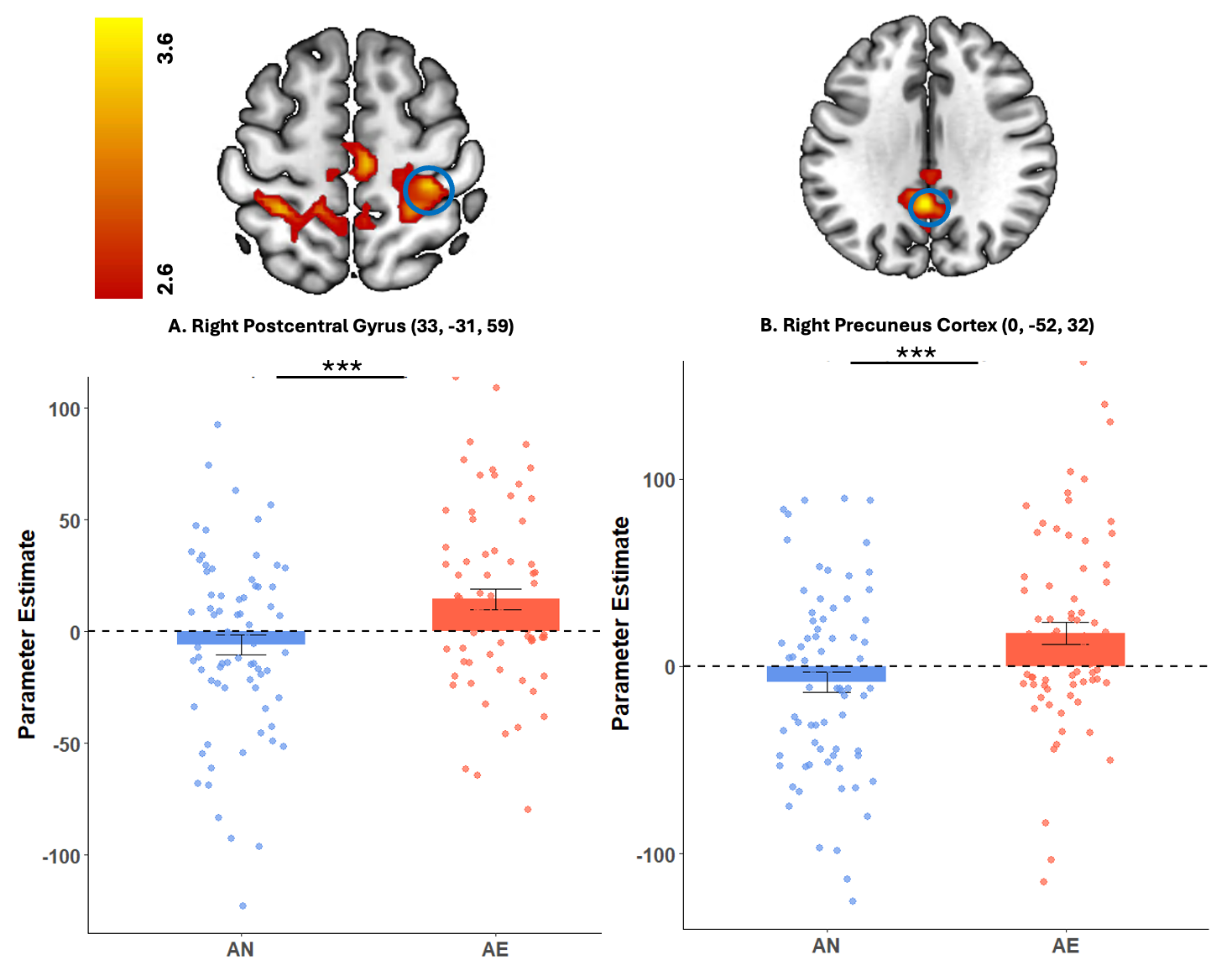


**Figure S1. Group Difference**

When removing youth who endorsed use at baseline and their matched AN pair, we found that that they differed in inhibitory control BOLD activation patterns. When compared to AN youth, AE youth displayed significantly higher activation in the right postcentral gyrus (Panel A) and the right precuneus cortex (Panel B) during the correct stop vs correct go contrast. The color bar represents the voxel T value. Image orientation is neurological (i.e., Right = Right).

# **Controlling for Race/Ethnicity**

## **Methods**

As an additional supplement, we included race/ethnicity as a covariate, given that our participant sample was matched on this variable. This was in addition to the main covariates—puberty score, age, and sex—that we included in our primary analyses.

## **Results**

### **Group Differences**

Whole-brain analysis of the overall inhibitory control between AN and AE youth controlling for race/ethnicity revealed increased activation in the left isthmus cingulate cortex (MNI: 0, -49, 32; peak Z = 3.5, *p*_FWE_ = 0.033, 160 voxels) and the left paracentral lobule (MNI: -9, -22, 53; peak Z =3.5, *p*_FWE_ < 0.001, 569 voxels) in AE youth compared to AN youth **(Table S2)**.

| **Region** | **Side** | **p(FWE)** | **p(unc)** | **k** | **T** | **Z** | **x** | **y** | **z** |
| --- | --- | --- | --- | --- | --- | --- | --- | --- | --- |
| **All Participants** | | | | | | | | | |
| Superior Temporal Gyrus | R | 0.03 | 0.002 | 169 | 4.2 | 4.1 | 48 | -1 | 2 |
| Inferior Parietal Cortex | R | 0.034 | 0.002 | 164 | 4 | 3.9 | 54 | -61 | 26 |
| Caudal Anterior Cingulate Cortex | R | 0.002 | 0.000 | 293 | 4 | 3.9 | 9 | 26 | 26 |
| Fusiform Gyrus | R | 0.000 | 0.000 | 824 | 3.8 | 3.7 | 12 | -85 | -4 |
| Precuneus Cortex | L | 0.000 | 0.000 | 466 | 3.6 | 3.5 | -12 | -55 | 50 |
| **AE > AN** | | | | | | | | | |
| Isthmus Cingulate Cortex | L | 0.033 | 0.002 | 160 | 3.6 | 3.5 | 0 | -49 | 32 |
| Paracentral Lobule | L | 0.000 | 0.000 | 466 | 3.6 | 3.5 | -9 | -22 | 53 |

**Table S2: Inhibitory Control Brain Activity**.

Inhibitory control brain activity (Correct Stop > Correct Go) across all participants, within AE youth and between group differences removing participants who used at baseline and their matched counterparts.

# **Controlling for Site at the ROI-level**

## **Methods**

As an additional supplement, we included assessment site as a covariate at the ROI level. We leveraged the ROI estimates obtained from Marsbar and built a mixed-effect logistic regression model including both brain regions and the main covariates—puberty score, age, and sex as fixed effects and scanner site as a random effect as previously suggested by ABCD.

## **Results**

| Variable | Estimate | P-value |
| --- | --- | --- |
| Isthmus Cingulate Cortex (L) | 0.008 | 0.074 |
| Paracentral Lobule (R) | 0.012 | 0.031 |
| Age | 0.023 | 0.389 |
| Pubertal Status | -0.019 | 0.951 |
| Biological Sex (Female) | 0.211 | 0.630 |

**Table S3.** Regression analyses predicting low-level alcohol experimentation. Models include primary predictors (isthmus cingulate cortex, paracentral lobule) and covariates (age, pubertal status and biological sex). This analysis accounted for scanner effects as a random effect.

# **Inclusion of Social Characteristics as Confounding Variables**

|  | Alcohol Experimentation | | | | | | |
| --- | --- | --- | --- | --- | --- | --- | --- |
|  | **Alcohol -Naïve** | | | **Alcohol-Exposed** | | |  |
|  | *N* | *Mean* | *SD* | *N* | *Mean* | *SD* | *Test* |
| **Peer Drug Use** | 80 |  |  | 80 |  |  | X_2_=0 |
| None | 76 | 95% |  | 76 | 95% |  |  |
| One or More | 4 | 5% |  | 4 | 5% |  |  |
| **Weight Discrimination** | 80 |  |  | 80 |  |  | X_2_=0 |
| No | 75 | 93.80% |  | 76 | 95% |  |  |
| Yes | 5 | 6.20% |  | 4 | 5% |  |  |
| **Perceived Drug Availability** | 80 | 0.0272 | 1.09 | 80 | 0.0326 | 0.949 | F=0.001 |
| **Parent's ADHD Symptoms** | 80 | -0.117 | 0.957 | 80 | 0.113 | 1.02 | F=2.151 |
| **Prenatal Drug Exposure** | 80 |  |  | 80 |  |  | X_2_=0.219 |
| No | 68 | 85% |  | 71 | 88.80% |  |  |
| Yes | 12 | 15% |  | 9 | 11.20% |  |  |
| **Economic Insecurities** | 78 | 0.615 | 1.42 | 80 | 0.45 | 1.24 | F=0.61 |
| **Alcohol Use Rule** | 80 |  |  | 80 |  |  | X_2_=0.031 |
| Permissive/No Rules | 22 | 27.50% |  | 24 | 30% |  |  |
| Strict Rules | 58 | 72.50% |  | 56 | 70% |  |  |

**Table S4.** Social characteristics related to alcohol use experimentation differentiating between alcohol-naïve and alcohol-exposed youth

# **Correlations with Behavior and Impulsivity Traits using ROI Masks**

To maintain relevancy to the inhibitory-control related regions, we conducted supplementary analyses in which we restricted the correlation analyses for brain activation with behavioral and personality measures to the inhibitory control regions identified from the whole brain analyses across all participants (3.3.1.1.). After applying the threshold applying a cluster-defining threshold of p < 0.005, pFWE < 0.05, no significant clusters survived.
